# Spectral analysis of layered sites using shaking table tests

**DOI:** 10.1371/journal.pone.0212766

**Published:** 2019-03-12

**Authors:** Gang Fan, Jianjing Zhang, Jinbiao Wu, Kongming Yan

**Affiliations:** 1 College of water resource and hydropower, Sichuan University, Chengdu, P. R. China; 2 School of Civil Engineering, Southwest JiaoTong University, Chengdu, China; 3 Priority Research Centre for Geotechnical Science and Engineering, Faculty of Engineering and Built Environment, The University of Newcastle, Newcastle, Australia; Northeastern University, UNITED STATES

## Abstract

The dynamic response analysis of horizontal and inclined layered sites using large-scale shaking table tests in various directions, including the dip direction, strike, vertical direction, slope direction and direction perpendicular to the interface of layered sites is conducted in this study. The Fourier spectrum and response spectrum characteristics in the horizontal site are first investigated in this study, and the dynamic responses of the inclined layered sites are then studied and compared to the corresponding responses of the horizontal layered site. The influence of dip angle on the response spectrum is also studied.

## Introduction

Construction is usually built on layered sites, including horizontal layered sites and inclined layered sites. A remarkable amount of researches regarding the dynamic response of horizontal layered sites have been carried out, and the results have been widely accepted [1][2][3][4][5][6]. Nevertheless, there is little relevant literature on the dynamic responses of inclined layered sites with a small dip angle. Furthermore, inclined layered sites are more common than horizontal layered sites. In recent years, layered slopes and landslides have been widely studied by different researchers [7][8][9][10], however, the geologic structure of layered slopes and landslides differs significantly from that of inclined layered sites, which will lead to great differences in dynamic response characteristics. Consequently, the research results on layered slopes and landslides cannot be directly used to analyse the dynamic responses of layered sites. Because the relevant research concentrating on the seismic characteristic differences between horizontal and small dip angle inclined layered sites is rare, it is difficult to estimate the dynamic response of small dip angle inclined layered sites using the relevant research results of horizontal layered sites. Thus, the study on the seismic characteristic differences between horizontal and small dip angle inclined layered sites is important.

**Fig 1:**
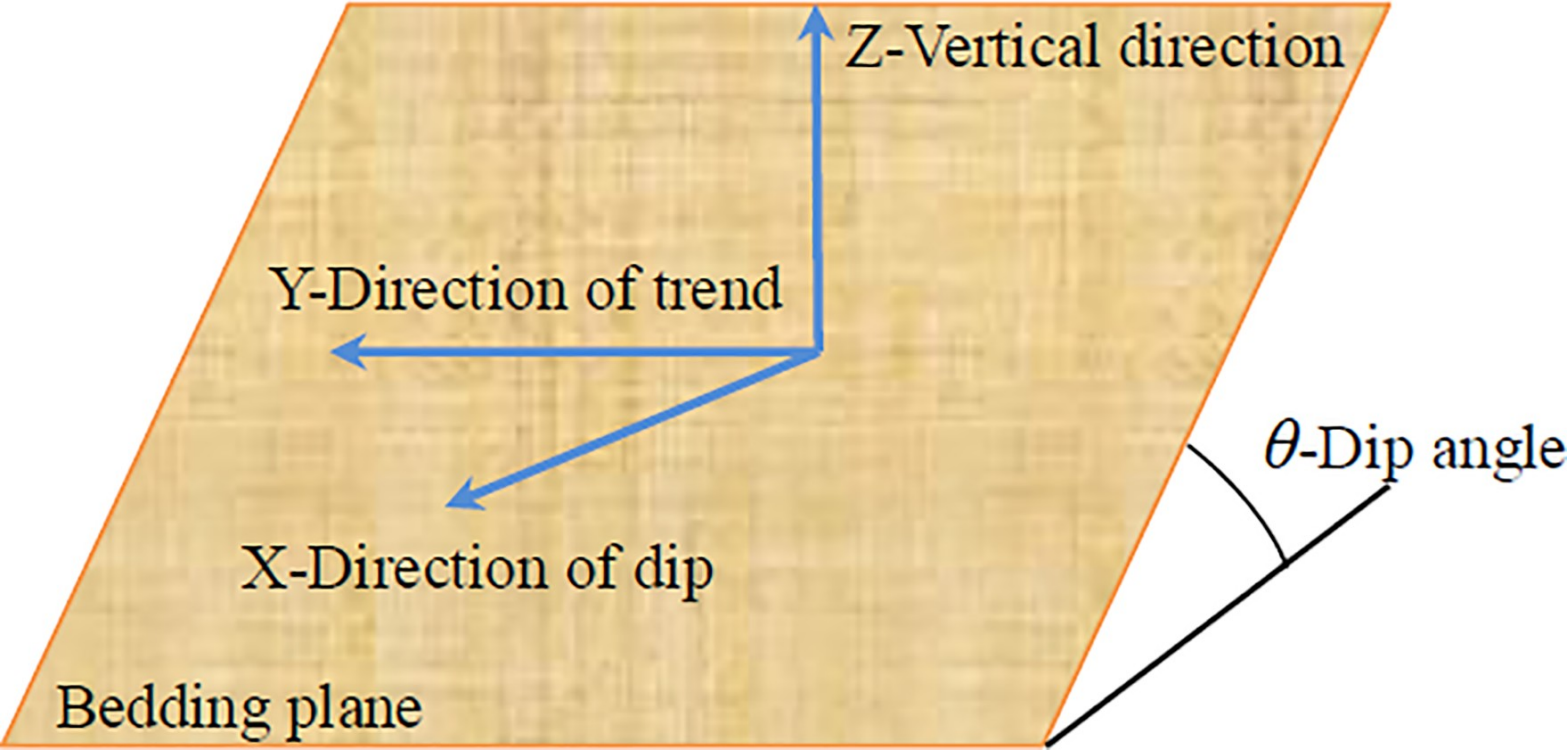
Coordinate in this analysis and the illustration of X, Y and Z directions.

As an approach to reveal the dynamic response characteristics directly, the large shaking table test has been widely employed by researchers to study the dynamic responses of slopes and earthquake-induced landslides [11][12][13][14][15]. Four layered site models with dip angles of 0°, 7.5°, 10° and 12.5° were developed in the same rigid box in this study. Recorded seismic waves with different amplitudes were used to excite the test model from the X, Y, and Z directions, which denote the dip direction, strike of the bedding plane and vertical direction, respectively, as illustrated in Fig 1. Here the vertical direction is defined as the direction perpendicular to the horizontal plane. The spectral characteristics of horizontal and inclined layered sites were studied, and the influence of dip angle on spectral characteristics was also discussed.

## Shaking table test

### Shaking table test device

The shaking table test was carried out at the Nuclear Power Institute of China (NPIC). The shaking table device has six degrees of freedom, the shaking table size is 6 × 6 m, the carrying capacity is 600 KN, the maximum lateral displacement is ±150 mm, the maximum vertical displacement is ±100 mm, the maximum acceleration under a full loading condition is 1.0 g horizontally and 0.8 g vertically, and the loading frequency range is 0.1–80 Hz.

### Similarity law

Owing to the complexity of soil mass and the limitation of test technology, it is difficult to comprehensively meet all similarity laws between the model and prototype in the experimental process. However, the similarity law can be established based on the consideration of the main parameters. According to the Buckingham *π* theorem, the similitude ratio of this shaking table test is 10 for length, 1 for density, 1 for acceleration, 3.16 for time, 0.316 for frequency and 10 for elasticity modulus[16]. A detail derivation process on the similitude ratio can be found in our previous study [17].

### Test model

**Fig 2:**
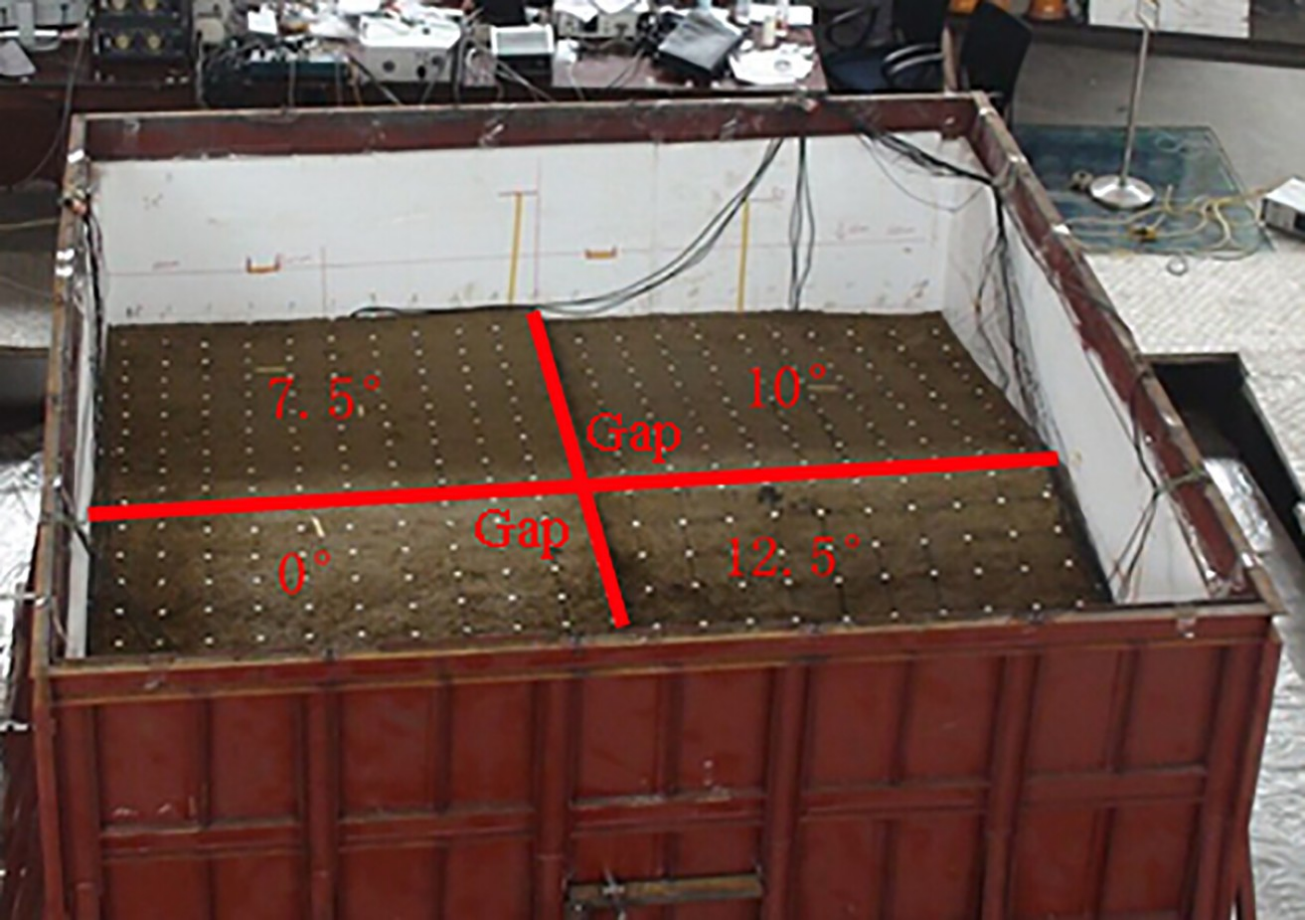
Rigid box and test model of layered sites.

**Fig 3:**
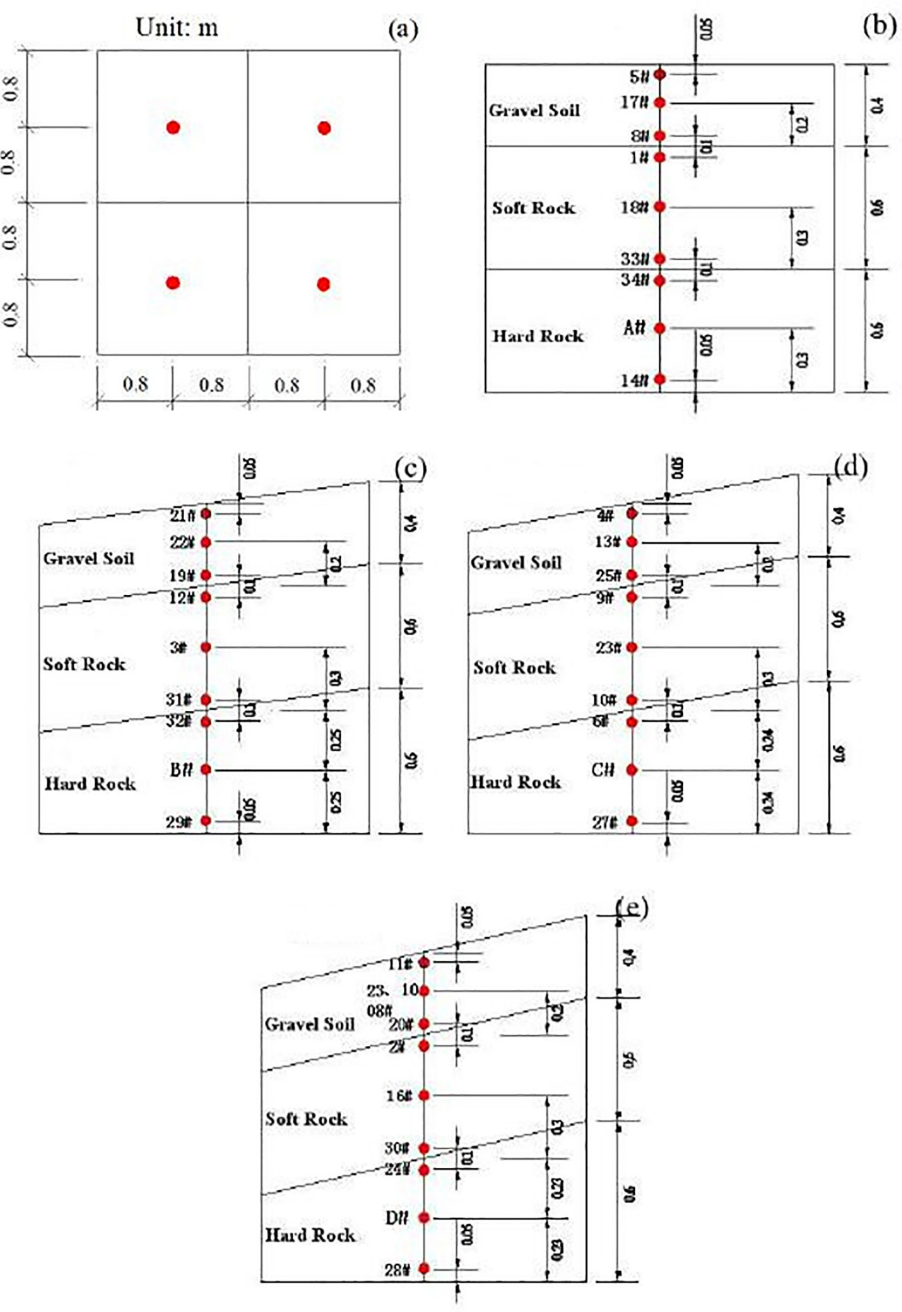
Location, direction and number of accelerometers (red points) in the shaking table test (unit: m), (a) layout plan, (b) horizontal layered site, (c) 7.5° inclined layered site, (d) 10° inclined layered site and (e) 12.5° inclined layered site.

The test model was constructed in a rigid 5.0 m × 5.0 m × 2.0 m box. The four test models with different dip angles were made in the same rigid box, and each model was isolated with gaps from surface to bottom of test model to eliminate interference with each other, as shown in Fig 2. The gaps were built in the process of model construction. Though the “model box effect” is inevitable in shaking table test [18][19][20], shaking table test has been widely adopted around the word [21][22][23][24]. However, based on our previous studies, the “model box effect” does not affect the regularity of analysis results and the test results are acceptable [14][15][25]. Lining foam with thickness of 10 cm was used as absorbing material to reduce the boundary reflection of the input seismic wave. The simulated strata of the model from top to bottom are gravel soil, soft rock and hard rock, respectively. The thickness of each stratum and dimension of the test model are shown in Fig 3.

The simulation materials were mixed according to the mix proportion obtained in previous laboratory experiments. Each model was constructed layer by layer, the thickness of each layer was 10 cm in the process of model construction. As the density control is a key point in the shaking table test, the creation of each layer was controlled by the model density, i.e., according to the model density and thickness of each layer, a certain quality simulation materials would be placed into the model box and tamped to a thickness of 10 cm. To mitigate the influence of construction, after one layer was made, its surface would be coarsened to connect well with the upper layer, and the next layer could then be made.

**Fig 4:**
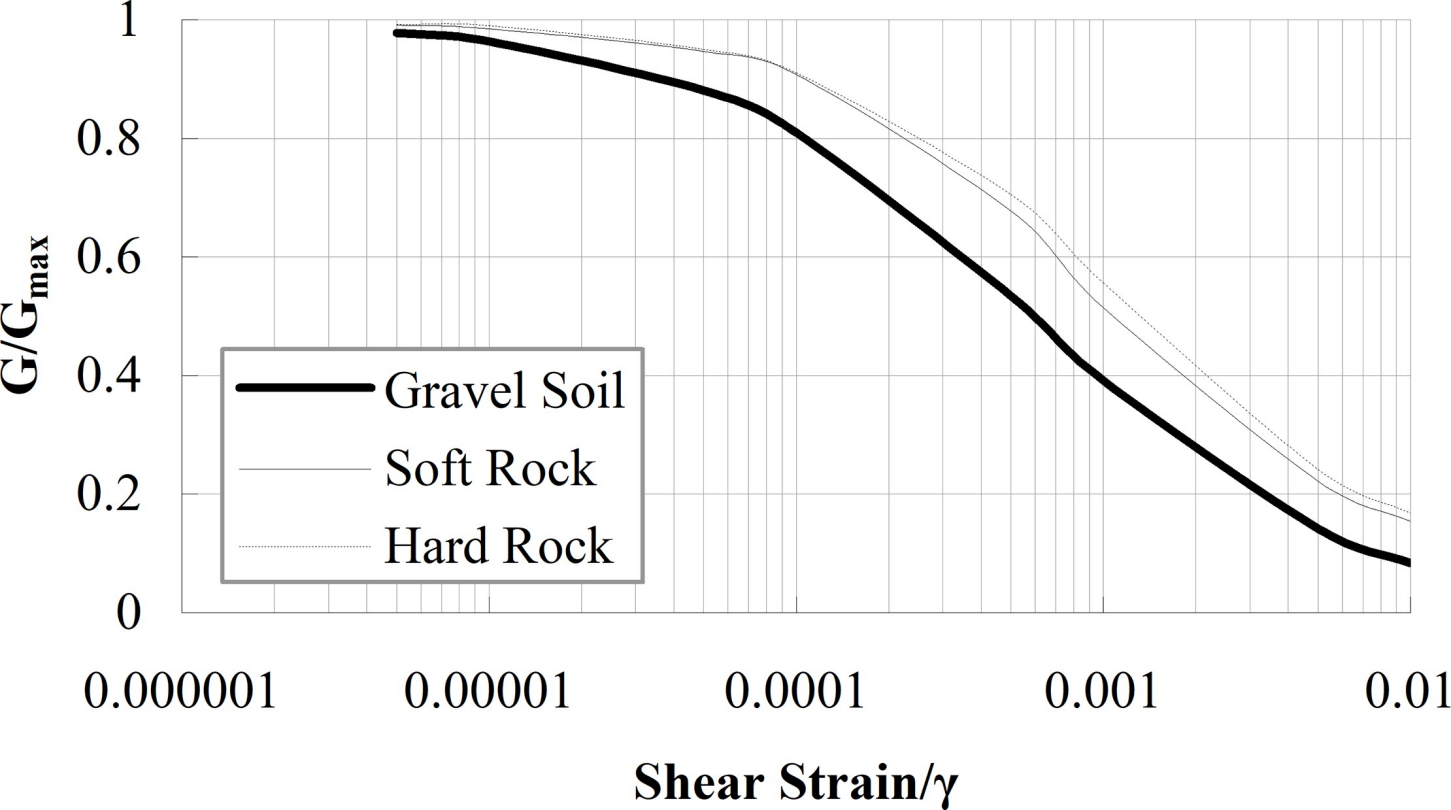
The curve of *G/Gmax* vs shear strain *γ*.

**Fig 5:**
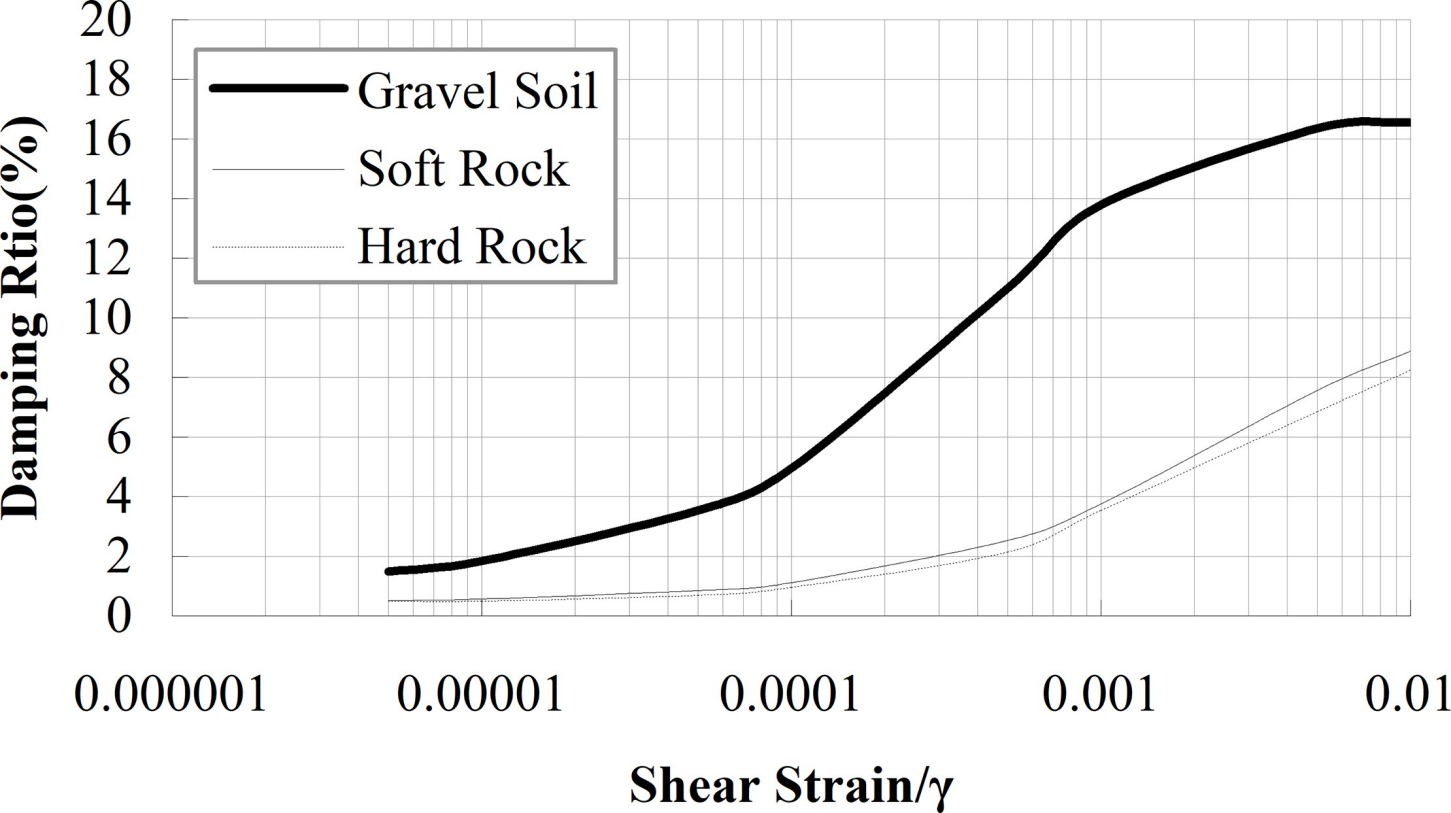
The curve of damping ratio *D* vs shear strain *γ*.

**Fig 6:**
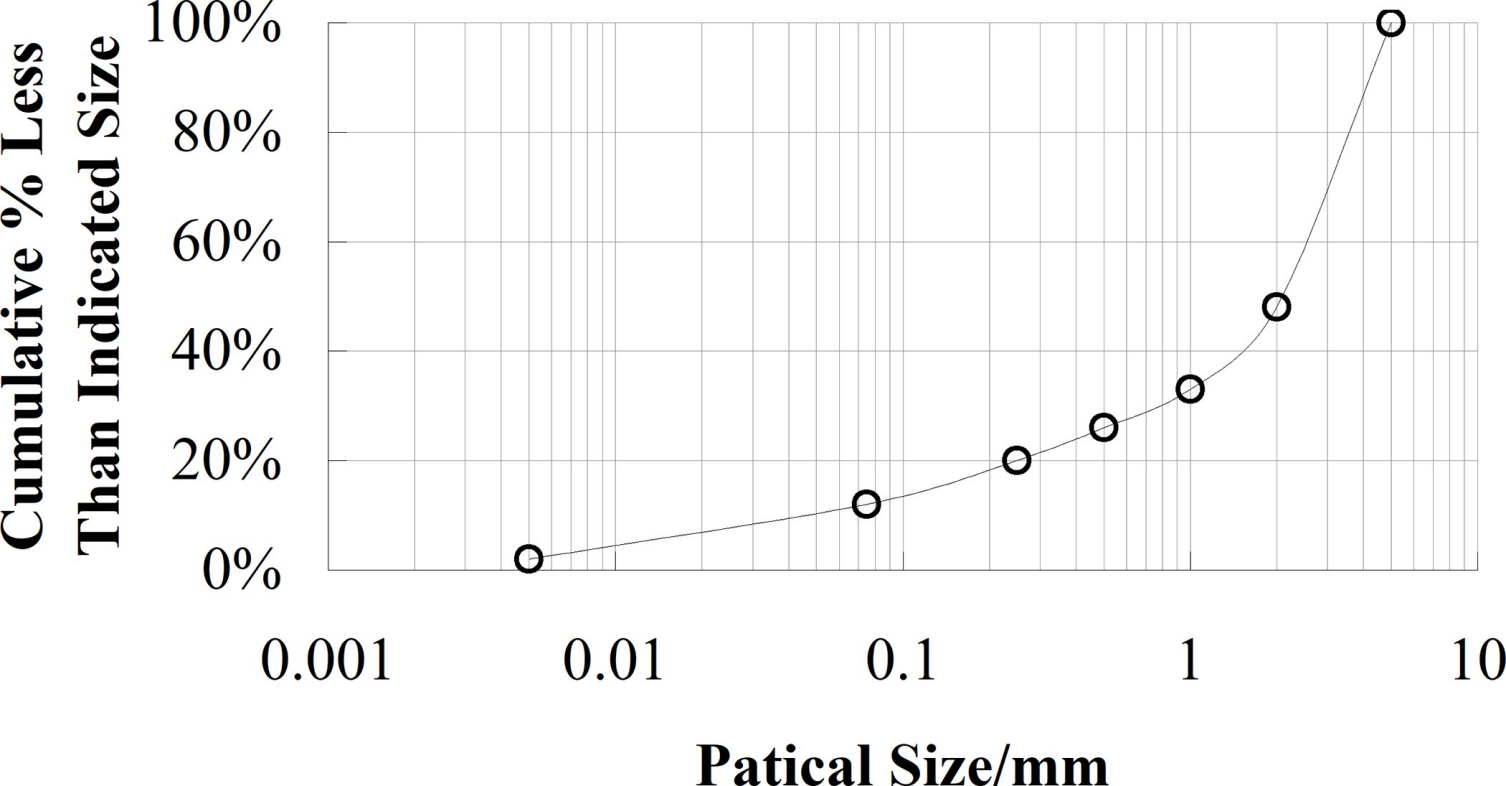
Particle size distribution curve of gravel soil.

After the test model was completed, a series of samples were taken. The direct shear test was performed to obtain the cohesive force (*c*) and internal friction angle (*φ*). The uniaxial compression test was performed to gain the elasticity modulus (*E*) and passion ratio (*μ*), the resonant column tests were performed to obtain the curves of *G/G*_*max*_*-γ* and *D-γ*, as shown in Figs 4 and 5. Here, *G* is the shear modulus, *G*_*max*_ is the maximum shear modulus, *D* is the damping ratio, and *γ* is the shear strain. The particle size distribution curve of the gravel soil is shown in Fig 6. A series of cylindrical samples (with diameter 280 mm and height 300 mm) were taken from the test model, the shear wave velocity of the material in each layer was obtained by the bender element method. The physical parameters of simulation materials are listed in Table 1.

**Table 1:** Physical parameters of the simulation materials.

*Strata*	*Density* *ρ* (kg/m^3^)	*Cohesive c* (kPa)	*Friction* *Angle* *φ* (°)	*Moisture Content* *ω* (%)	*Elastic Modulus* *E* (MPa)	*Poisson Ratio* *μ*	*Shear Wave Velocity*	*Mix Proportion*
Gravel Soil	1900	18	25	13.95	1.5	0.16	92	sand: clay: silica sand: water = 33:35:18:12
Soft Rock	2200	104	41	7.83	2100	0.25	254	sand: clay: gypsum: water = 75:20:20:9
Hard Rock	2300	200	45	9.66	2780	0.20	362	sand: clay: gypsum: water: blanc fixe = 5:3:2.5:1.4:4

### Accelerometer layout

Because this study focuses primarily on spectral analysis of layered sites, the accelerations in the models were monitored by accelerometers. In recent studies, some common types of accelerometers have been used, including the mechanical magneto electric type, piezoelectric suspension type, electromagnetic suspension type and optical grating type [26]. The optical grating accelerometer has the highest sensitivity among all types of accelerometers mentioned above [27]. Piezoelectric suspension accelerometers were used in this shaking table test to monitor the acceleration. The sensitivity of the accelerometer is 173.46 mv/g in the horizontal direction and 192.08 mv/g in the vertical direction. Accelerometers were set at the shaking table board, the middle of each stratum and the points 5 cm above and below each interface. The layout and serial number of each accelerometer in the test model are shown in Fig 3.

In this paper, a horizontal layered site is defined as in Fig 3(B), and an inclined layered site is defined as in Fig 3(C), 3(D) and 3(E).

### Test loading

**Fig 7:**
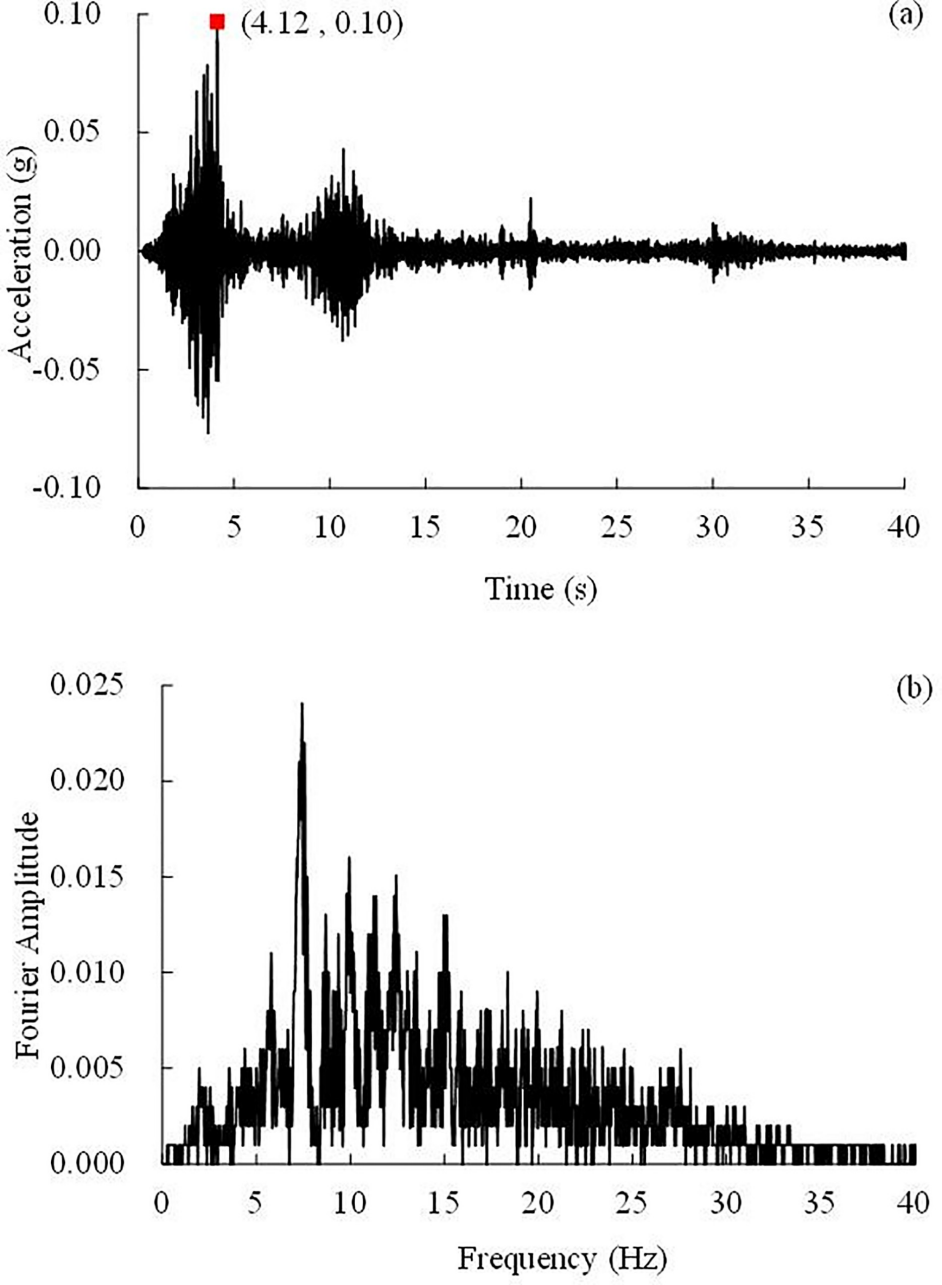
0.10 g WenChuan earthquake wave and Fourier spectrum, (a) Time history, (b) Fourier spectrum.

**Fig 8:**
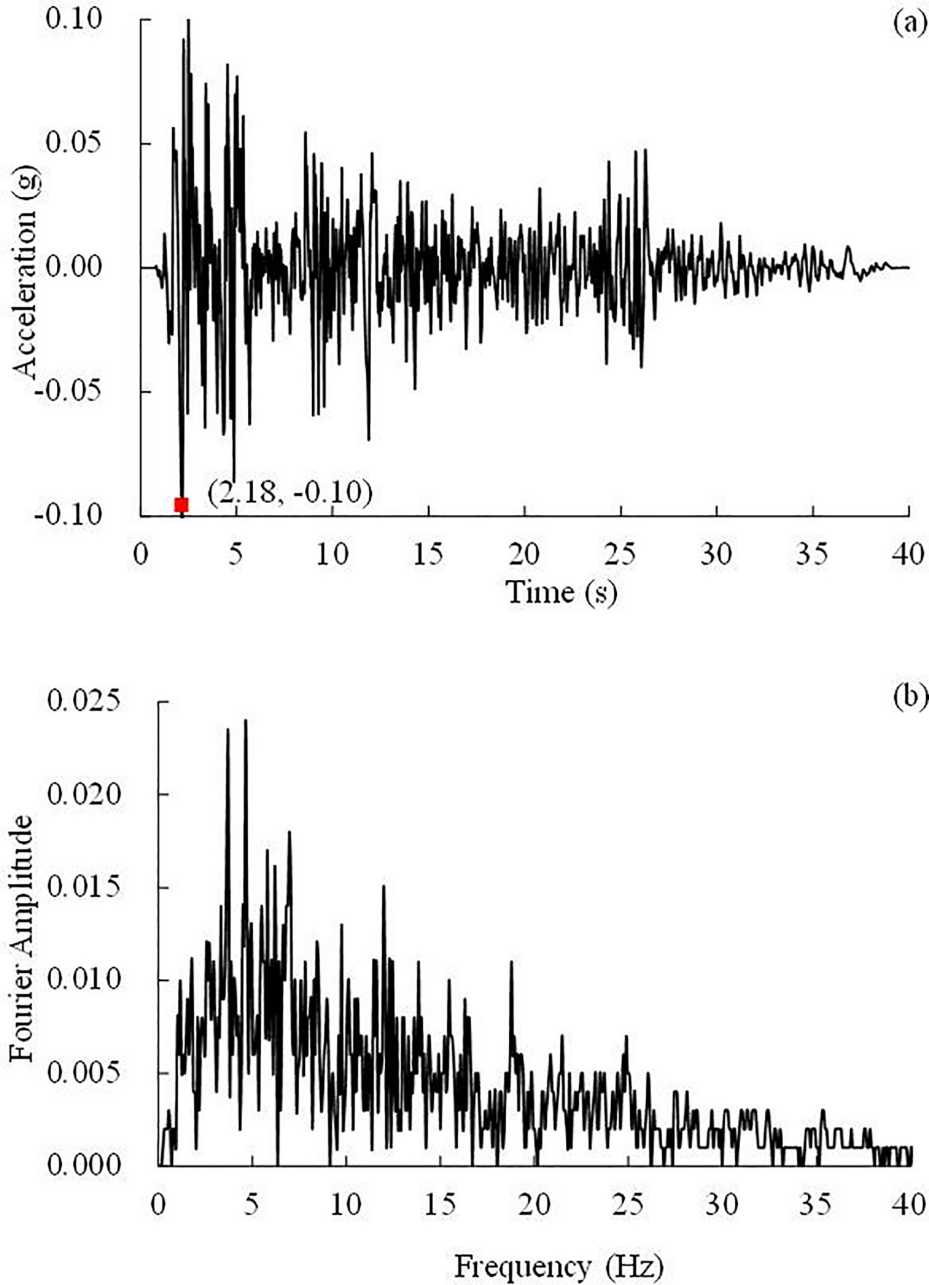
**0.10 g El Centro earthquake wave and its Fourier spectrum:** (a) Time history, (b) Fourier spectrum.

**Fig 9:**
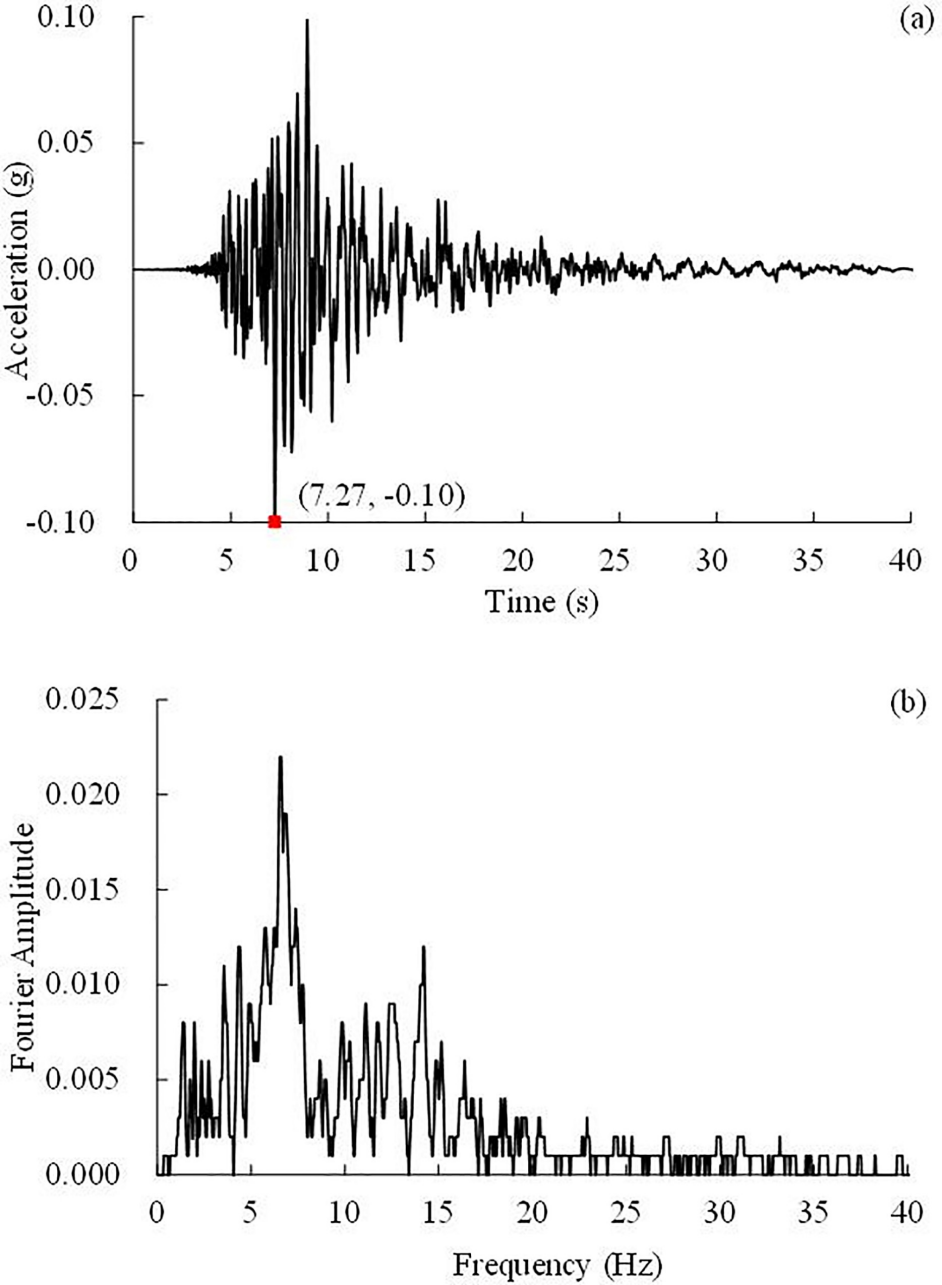
**0.10 g Kobe earthquake wave and its Fourier spectrum:** (a) Time history, (b) Fourier spectrum.

The Kobe earthquake record, El Centro earthquake record and WenChuan earthquake record were used to excite the test model in the shaking table tests from the X, Y, and Z directions. The basic information of these records is listed in Table 2. The peak accelerations of the input seismic waves were adjusted to 0.10 g, 0.20 g, 0.32 g, 0.40 g and 0.50 g, respectively. These adopted acceleration time histories are scaled in the time domain, the time axis is scaled based on the time similitude ratio. The 0.10 g horizontal input earthquake and their Fourier spectra are shown in Figs 7, 8 and 9.

**Table 2:** Basic information of adopted earthquake records.

*Earthquake*	*Time*	*Magnitude*	*Focal depth (km)*	*Station code*
Kobe	17/01/1995	*M*_w_ 6.9	17	JMA, Japan
El Centro	18/05/1940	*M*_w_ 6.9	24	El Centro, USA
WenChuan	12/05/2008	*M*_w_ 7.9	14	Wolong, China

## Spectral analyses of horizontal layered site

### Fourier spectra analysis in horizontal layered site

**Fig 10:**
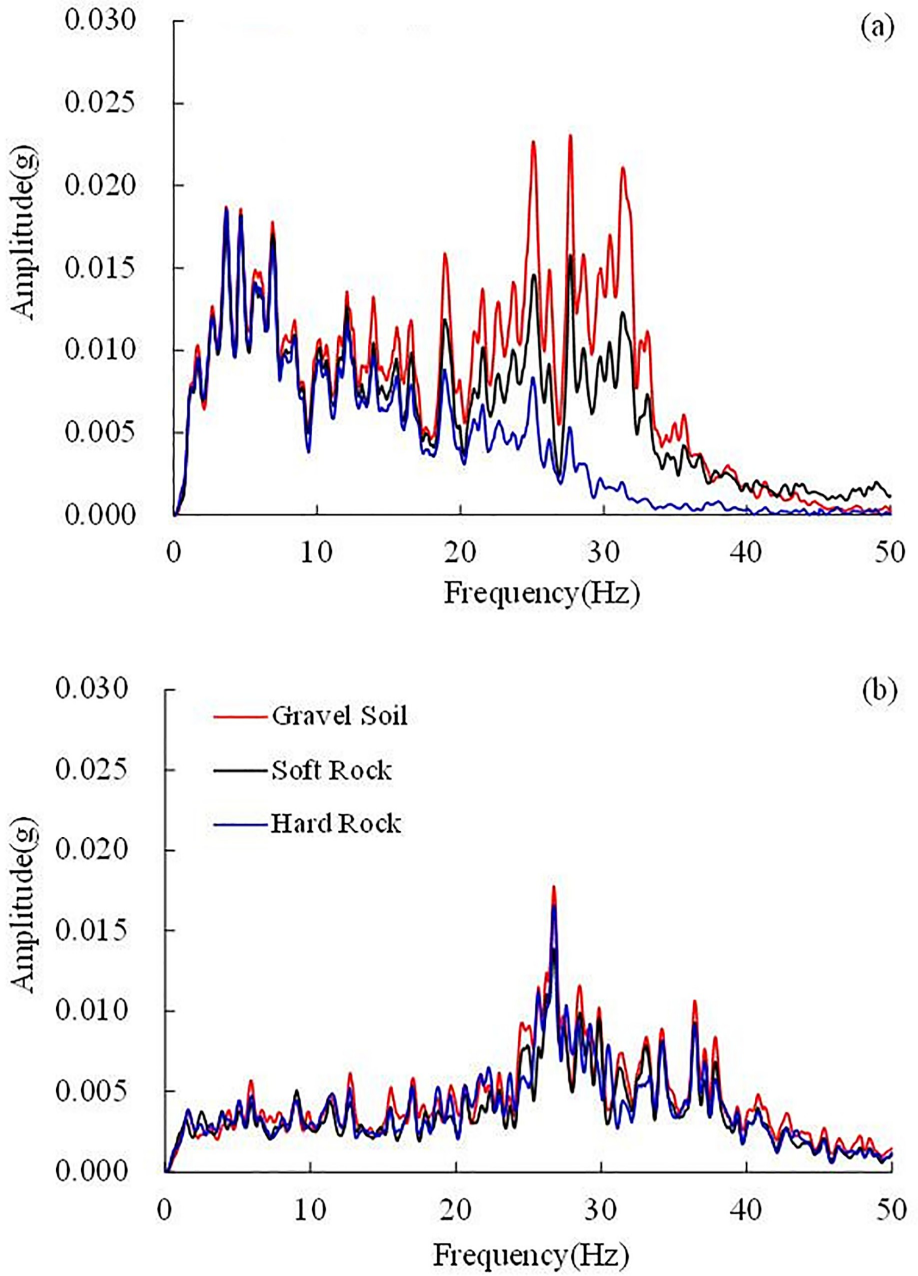
The Fourier spectra of different layers under the 0.10 g El Centro earthquake record, (a) in the horizontal direction (i.e. X direction) and (b) in the vertical direction (i.e. Z direction).

**Fig 11:**
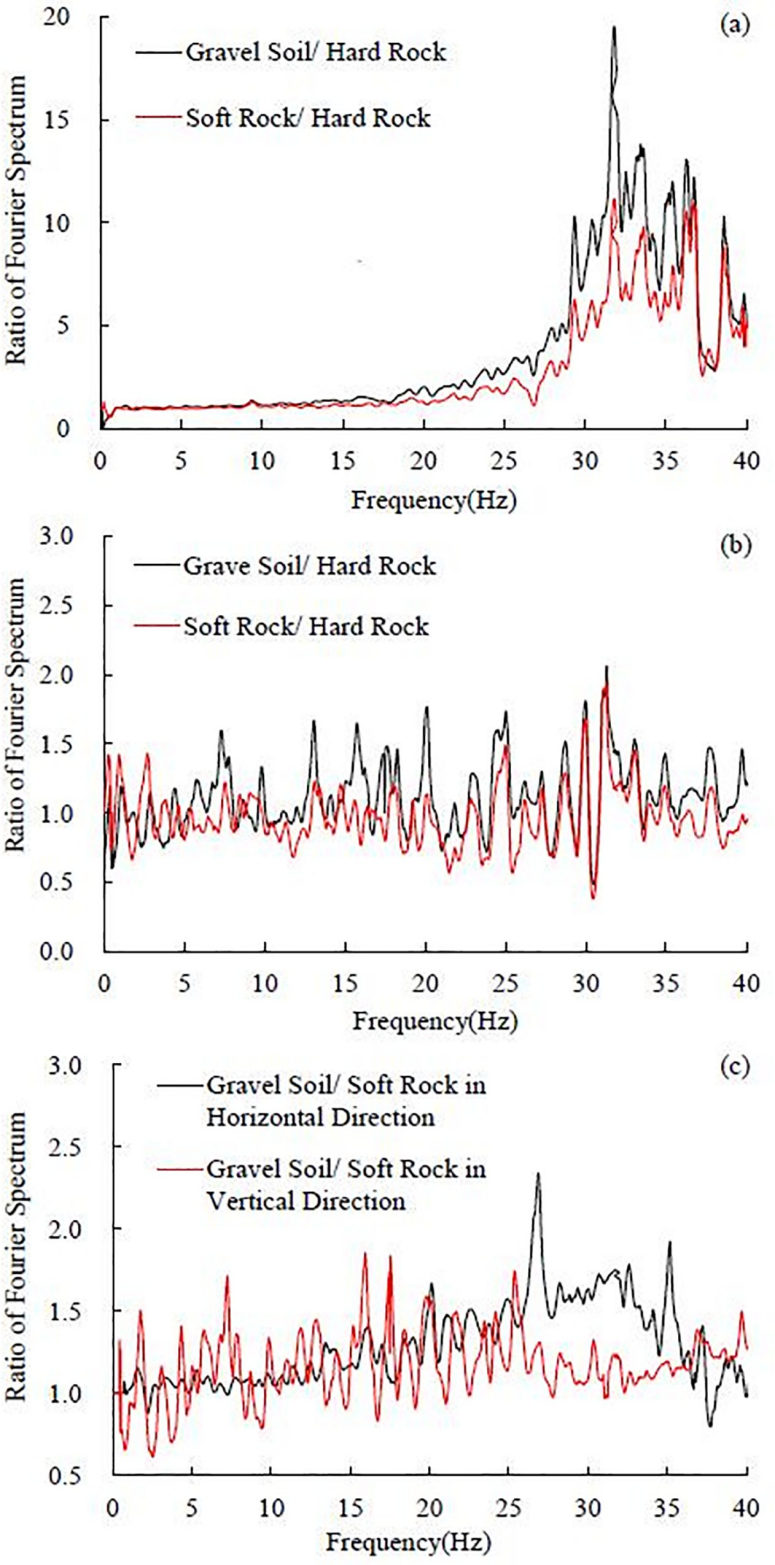
The ratio of Fourier spectra under 0.10 g El Centro earthquake record in the horizontal site, (a) gravel soil layer and soft rock layer to hard rock layer in the horizontal direction, (b) gravel soil layer and soft rock layer to hard rock layer in the vertical direction and (c) gravel soil layer to soft rock layer in the horizontal and vertical directions.

The Fourier spectra of each stratum (i.e., gravel soil layer, soft rock layer, hard rock layer) in the horizontal layered site under the 0.1 g El Centro earthquake record were calculated based on the accelerometers at the top of each stratum, i.e., 5# accelerometer in the gravel soil layer, 1# in the soft rock layer and 34# in the hard rock layer. The Fourier spectra in the horizontal direction are shown in Fig 10(A), and the Fourier spectra in the vertical direction are shown in Fig 10(B). To reveal the change law of frequency components in the wave propagation from the hard rock layer to the gravel soil layer, the ratio of Fourier spectrum was defined as the ratio of the Fourier spectrum amplitude of a certain stratum to the Fourier spectrum amplitude of the hard rock layer. If the ratio of the Fourier spectrum is larger than 1, the frequency component will be amplified in the wave propagation, in the opposite case, the frequency component will be weakened. The ratios of the Fourier spectra in the horizontal and vertical directions are shown in Fig 11(A) and 11(B), respectively.

In the horizontal direction, the 0–13 Hz frequency components almost do not change, whereas the 13–40 Hz frequency components are amplified in a large scale, especially the 30–40 Hz frequency components. It is worth noting that the ratio of the Fourier spectrum in the gravel soil layer is larger than that in the soft rock layer. The maximal ratio of the Fourier spectrum in the gravel soil layer is 19.39, which is obtained at 31.84 Hz, the maximal ratio of the Fourier spectrum is 10.85 for the soft rock layer, which is obtained at 31.87 Hz. Huang et al. calculated Fourier spectral ratios at surface soil layer relative to the depths of 6, 11, 17 and 47 m [4]. The maximal spectral ratio is nearly 10, which is close to the maximal ratio in the soft rock layer and smaller than the maximal ratio in the gravel soil layer obtained in this test. A large variation of the spectral ratios between the surface and different depths during the strong ground motions was also observed by Aguirre and Irikura using the acceleration records of the 1995 Hyogo-ken Nanbu earthquake at Port Island, Kobe [6].

The resonance frequency *f* of the horizontal layered site can be estimated by the following equation [28][29]:

$$f=\frac{V}{4H}$$ (1)

where *f* is the resonance frequency, *H* is the total height of the horizontal layered site, and *V* is the average shear wave velocity given by:

$$V=\frac{\sum_{i=1}^{n}h_{i}}{\sum_{i=1}^{n}\frac{h_{i}}{v_{i}}}$$ (2)

where *h_i_* and *v_i_* are the thickness and the shear wave velocity of the layer, respectively, as shown in Table 3.

**Table 3:** Shear wave velocity and height of every layer.

*No*. *of layer*	*Name of layer*	*Shear wave velocity*	*Height (m)*
1	Gravel soil layer	92	0.4
2	Soft rock layer	254	0.6
3	Hard rock layer	362	0.6

The resonance frequency *f* of the horizontal layered site is 29.88 Hz based on Eqs (1) and (2). Fig 11(A) implies that the dominant frequency of the horizontal layered site is approximately 31 Hz, which is very close to the calculated resonance frequency (29.88 Hz). When the dominant frequency of the layer is close to the resonance frequency of the layered site, resonance effect can be observed. Hence, the significant amplification effect of frequencies (31.84 Hz and 31.87 Hz) in this study can be explained by the resonance effect of the horizontal layered site.

In the vertical direction, all frequency components hardly change in wave propagation, and the amplification effect of the frequency components is slight, as shown in Fig 11(B). The amplification effect of the frequency components in the gravel soil layer is slightly larger than that in the soft rock layer.

As seen in Fig 11 (C), the Fourier spectrum amplification between the gravel soil layer and the soft rock layer is not obvious compared to that between the gravel soil layer or soft rock layer and the hard rock layer. In the horizontal direction, the 20–35 Hz frequency components are amplified on a small scale, and the peak ratio of the Fourier spectrum is 2.32, which is obtained at 26.90 Hz. Other frequency components (i.e. 0–20 Hz and 35–40 Hz) in the horizontal direction and all frequency components in the vertical direction are almost not amplified between the gravel soil layer and the soft rock layer. It can be concluded according to the above analysis that the frequency components in the horizontal direction are mainly amplified between the soft rock layer and the hard rock layer, and the amplification between the gravel soil layer and the soft rock layer is slight.

**Fig 12:**
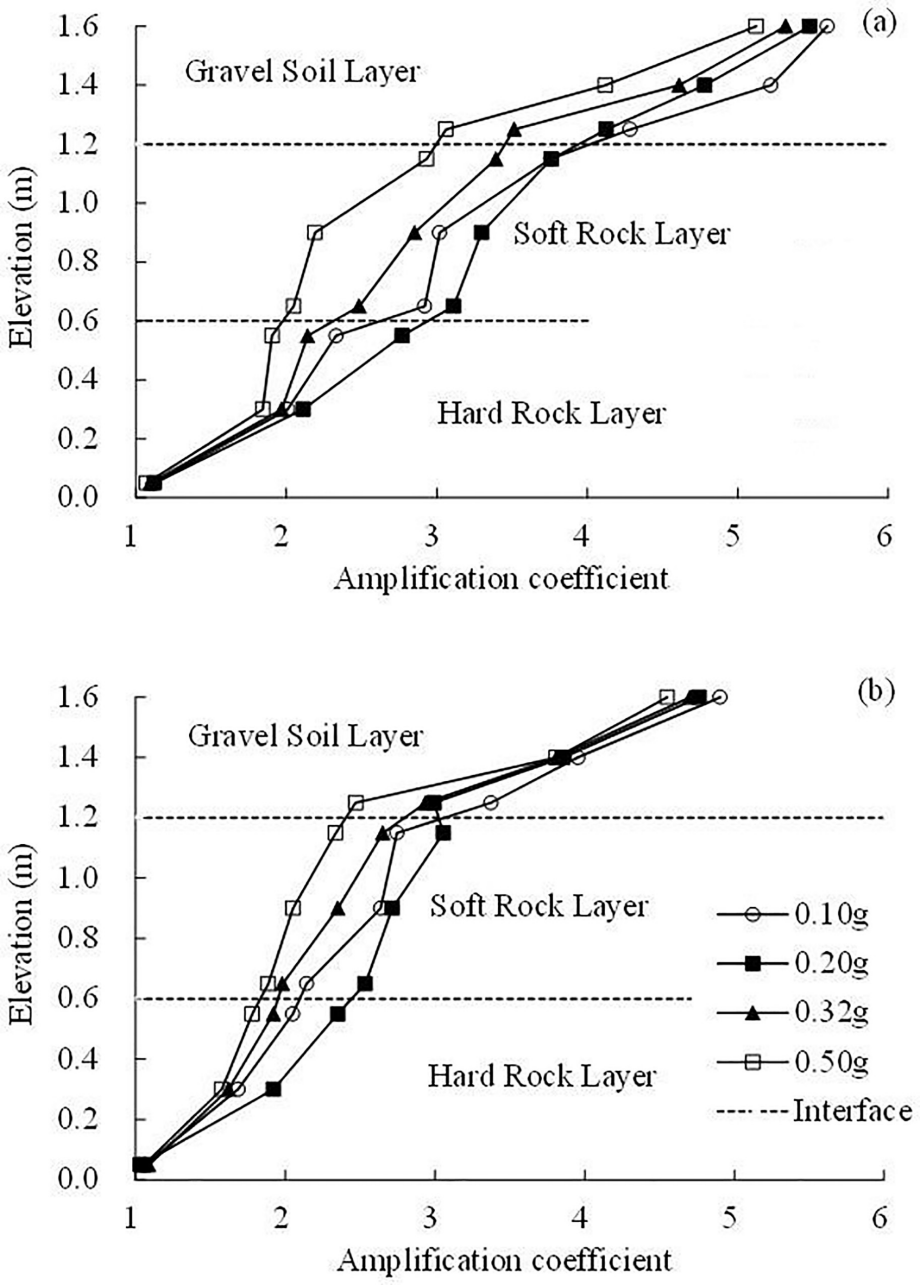
Acceleration amplification coefficient (a) under the El Centro earthquake record and (b) under the WenChuan earthquake record.

It can be concluded from the above analysis that in the horizontal site, the ratio of the Fourier spectrum in the gravel soil layer is larger than that in the soft rock layer in the horizontal direction, as illustrated in Fig 11(A) and 11(C), leading to the dynamic response of the gravel soil layer is stronger than that in the soft rock layer. An analysis of the acceleration amplification effect shows that the acceleration amplification coefficient in the gravel soil layer is larger than that in the soft rock layer, which conforms to the above analysis in frequency domain, as shown in Fig 12.

It is worth noting that in the gravel soil layer, the amplification coefficients decrease with increasing PGA, however, in the soft rock layer and hard rock layer, the higher amplifications are generally obtained for PGA = 0.20 g, whereas the lower ones are obtained for 0.50 g. This implies that the gravel soil layer begins to show nonlinear characteristics when PGA = 0.10 g, whereas the soft rock layer and hard rock layer begin to show nonlinear characteristics when PGA = 0.20 g. The reason for nonlinear response is that when the geotechnical material shear strain increases and shear modulus decreases with increasing PGA, the hysteresis curve will be fuller, and the dissipated shaking energy by the geotechnical material will increase, thus weakening the dynamic response of the site, therefore, the amplification coefficients decrease. The nonlinear response of the surface layer is also observed by Huang et al. in Taiwan [4] and Beresnev et al. [3]. The problem of nonlinear dynamic response was in the spotlight again when considerable discrepancy between the strong and weak motion amplification factors was reported by Chin and Aki for the epicentral zone of the Loma Prieta Earthquake [3]. Compared to the soft rock layer and hard rock layer, the gravel soil layer is easier to suffer large shear strain, hence the gravel soil layer shows nonlinear characteristics ahead of the soft rock layer and hard rock layer.

### Response spectrum analysis in horizontal layered site

**Fig 13:**
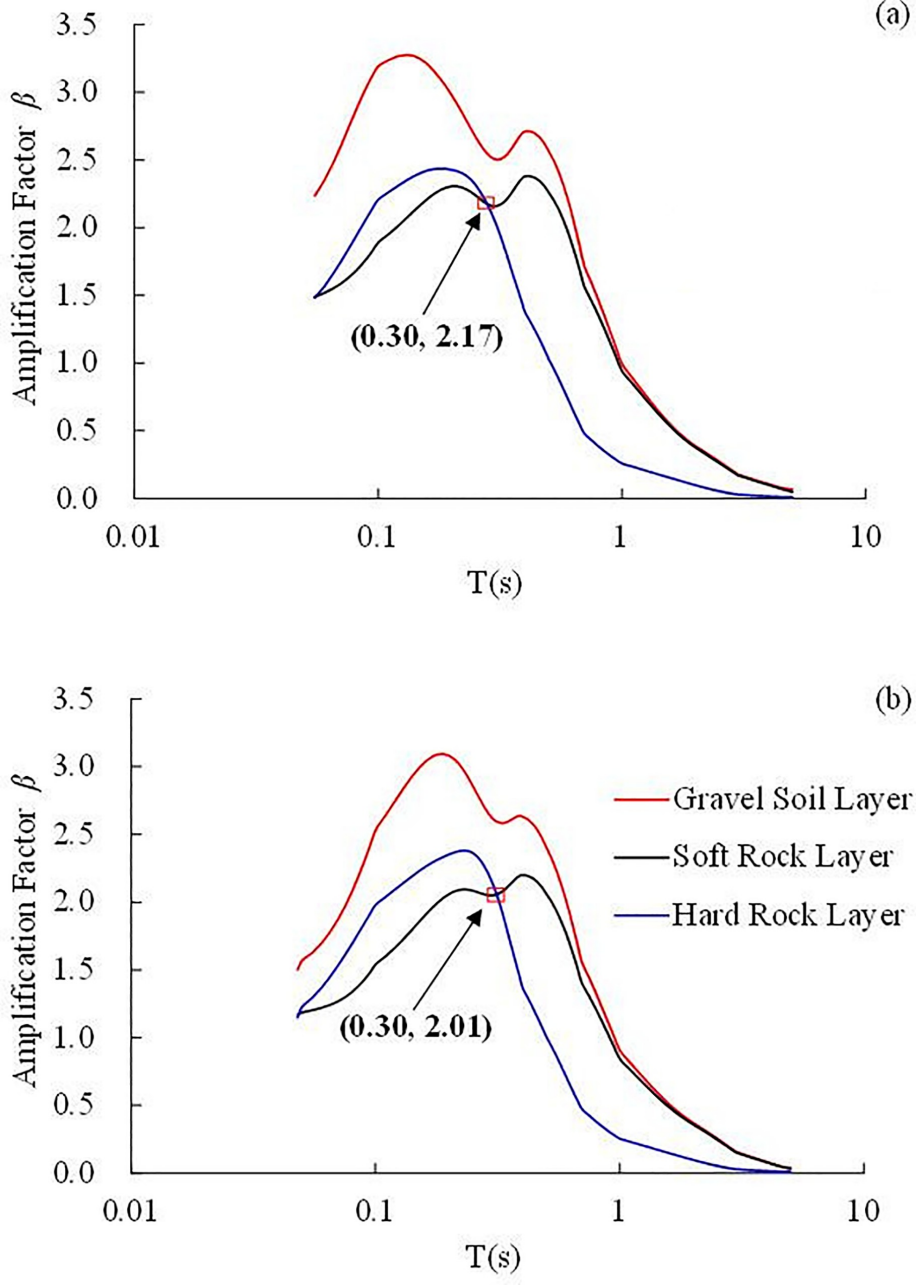
**Acceleration response spectrum under the Kobe earthquake record with different amplitude:** (a) 0.10 g, (b) 0.50 g.

The acceleration response spectra of a single-degree-of-freedom (SDOF) system with a 5% damping ratio under the Kobe earthquake record with amplitudes of 0.10 g and 0.50 g were calculated as shown in Fig 13. The response spectra of the gravel soil layer, soft rock layer and hard rock layer were calculated based on the time history of 5#, 34# and 1# accelerometer, respectively.

Fig 13 shows that the amplification factor *β* of the response spectra under the 0.50 g earthquake wave is close to that under the 0.10 g earthquake wave. Because of the significant amplification effect of the gravel soil layer, as shown in Fig 12, the amplification factor *β* of the response spectra of the gravel soil layer is larger than those of the soft rock layer and hard rock layer. In the short period (*T* ≤ 0.3 s, where *T* is the period), the sequence of the response spectrum amplification factor *β* is gravel layer > hard rock layer > soft rock layer. Whereas in the long period (*T* > 0.3 s), the sequence of the spectral response amplification factor is gravel layer > soft rock layer > hard rock layer. The result indicates that on the one hand, the buildings constructed on the gravel layer will suffer a stronger dynamic response than the soft rock layer and hard rock layer. On the other hand, with regard to the buildings constructed on the soft rock layer or hard rock layer, when the building period *T* ≤ 0.3 s, the buildings constructed on the hard rock layer will suffer a stronger dynamic response than those on the soft rock layer, whereas the building constructed on the soft rock layer will suffer a stronger dynamic response than those on the hard rock layer when the building period *T* > 0.3 s.

### Spectrum analysis of inclined layered site

A remarkable amount of research on the dynamic response of horizontal layered sites has been undertaken, and relevant research results have been widely applied to regional seismic safety evaluation and seismic design [1][2][4][30]. The dynamic spectrum characteristics of small dip angle inclined layered sites were analysed in this study, mainly including the Fourier spectrum and response spectrum. The ratio of the Fourier spectrum and the ratio of the response spectrum between the inclined layered sites and the horizontal layered site were used as a quantitative index in the following analysis.

The influence of dip angle on the Fourier spectrum and response spectrum are discussed in this study. The data employed to calculate the Fourier spectrum and response spectrum in this study were collected from the 5# accelerometer at the layered horizontal site, 21# at the 7.5° inclined layered site, 4# at the 10° inclined layered site and 11# at the 12.5° inclined layered site.

### Effect of dip angle on Fourier spectra

**Fig 14:**
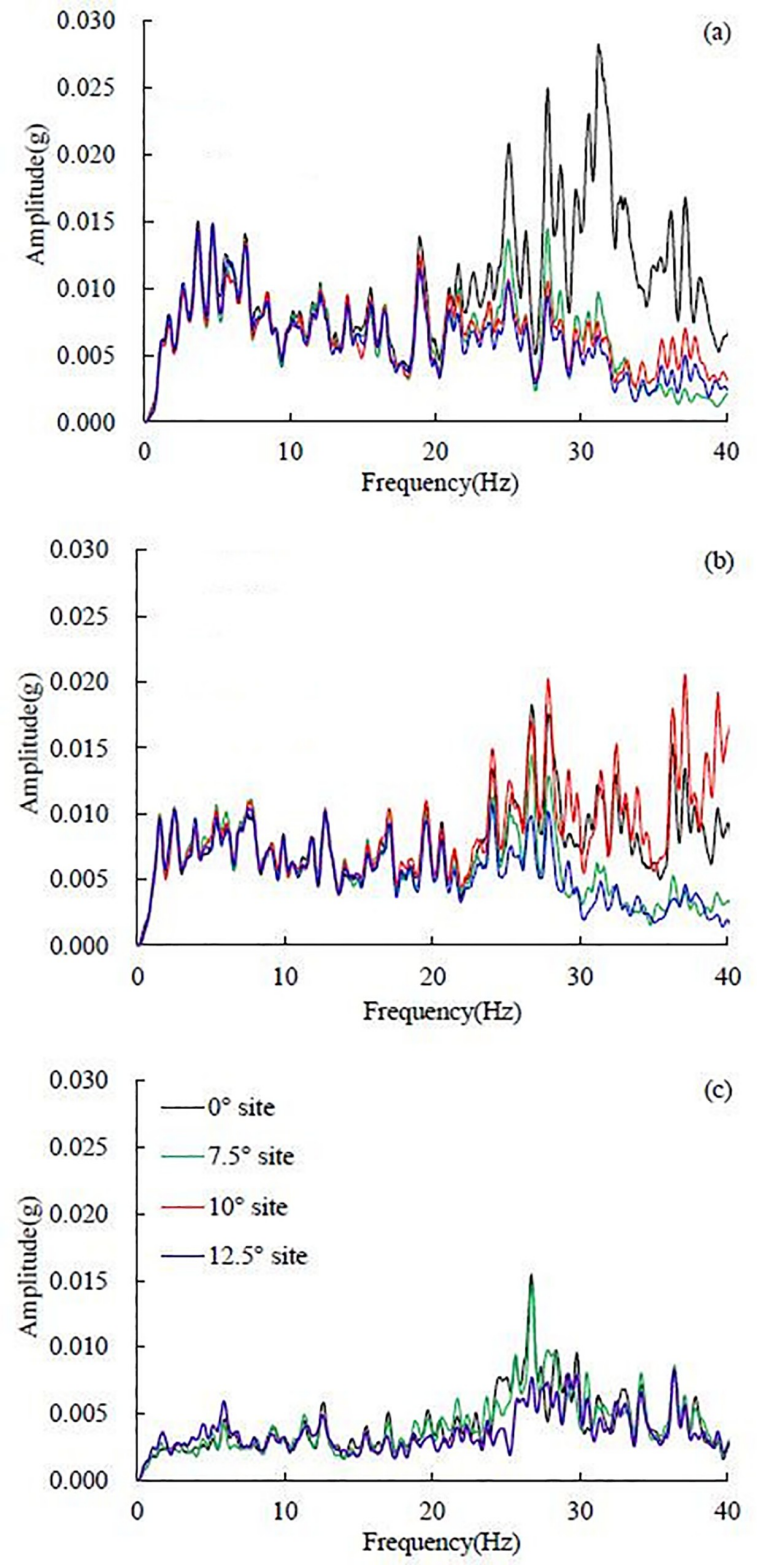
**The Fourier spectra under 0.1 g El Centro earthquake record:** (a) in the dip direction, (b) in the strike and (c) in the vertical direction.

**Fig 15:**
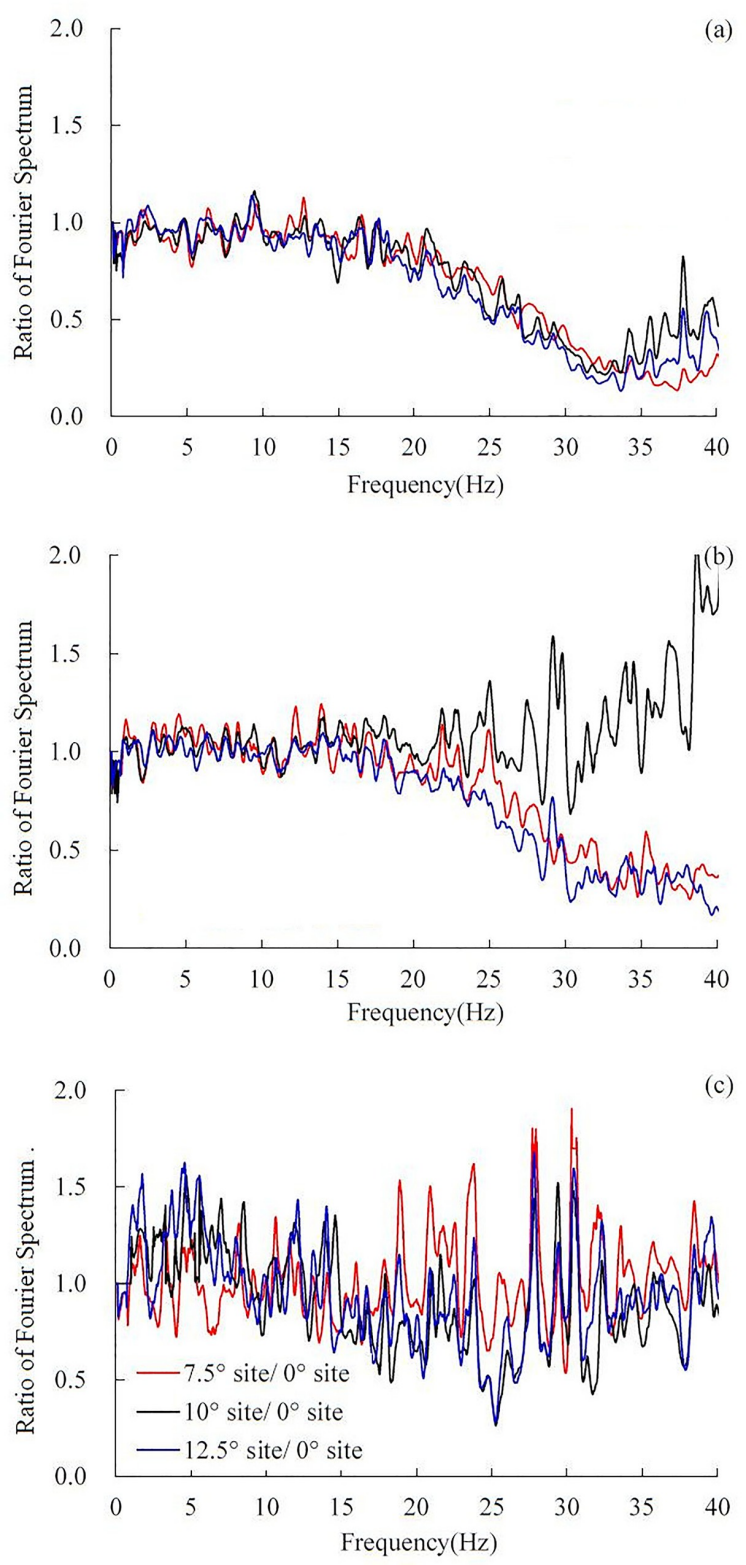
**Ratio of Fourier spectra under 0.1 g El Centro earthquake record:** (a) in the dip direction, (b) in the strike and (c) in the vertical direction.

To reveal the influence of dip angle on the frequency components, the Fourier spectra of the sites with different dip angles were calculated. The Fourier spectra in the dip direction, strike and vertical direction are shown in Fig 14(A), 14(B) and 14(C), respectively. The ratio of the Fourier spectrum, defined as the ratio of the Fourier spectrum amplitude of the inclined layered site to the Fourier spectrum amplitude of the horizontal layered site, is shown in Fig 15(A), 15(B) and 15(C).

In the dip direction, as seen in Fig 15(A), in the frequency band of 0–18 Hz, the ratio of the Fourier spectra is nearly 1, which indicates that the influence of dip angle on 0–18 Hz frequency components can be ignored. However, because the earthquake energy at high frequencies (18–40 Hz) will be dissipated by the inclined interface in the dip direction, the ratio of the Fourier spectrum is less than 1 in the frequency band of 18–40 Hz, that is to say, the 18–40 Hz frequency components of the inclined layered sites were weakened compared to the horizontal layered site. The reduction of these 3 inclined layered sites in the frequency band of 18–35 Hz are very close, whereas in the frequency band of 30–40 Hz, the reduction of the 10° inclined site is least. From this analysis, it can be concluded that in the dip direction, the dip angle has little influence on the 0–18 Hz frequency components, whereas the 18–40 Hz frequency components of the inclined layered sites were weakened compared to the horizontal layered site.

**Fig 16:**
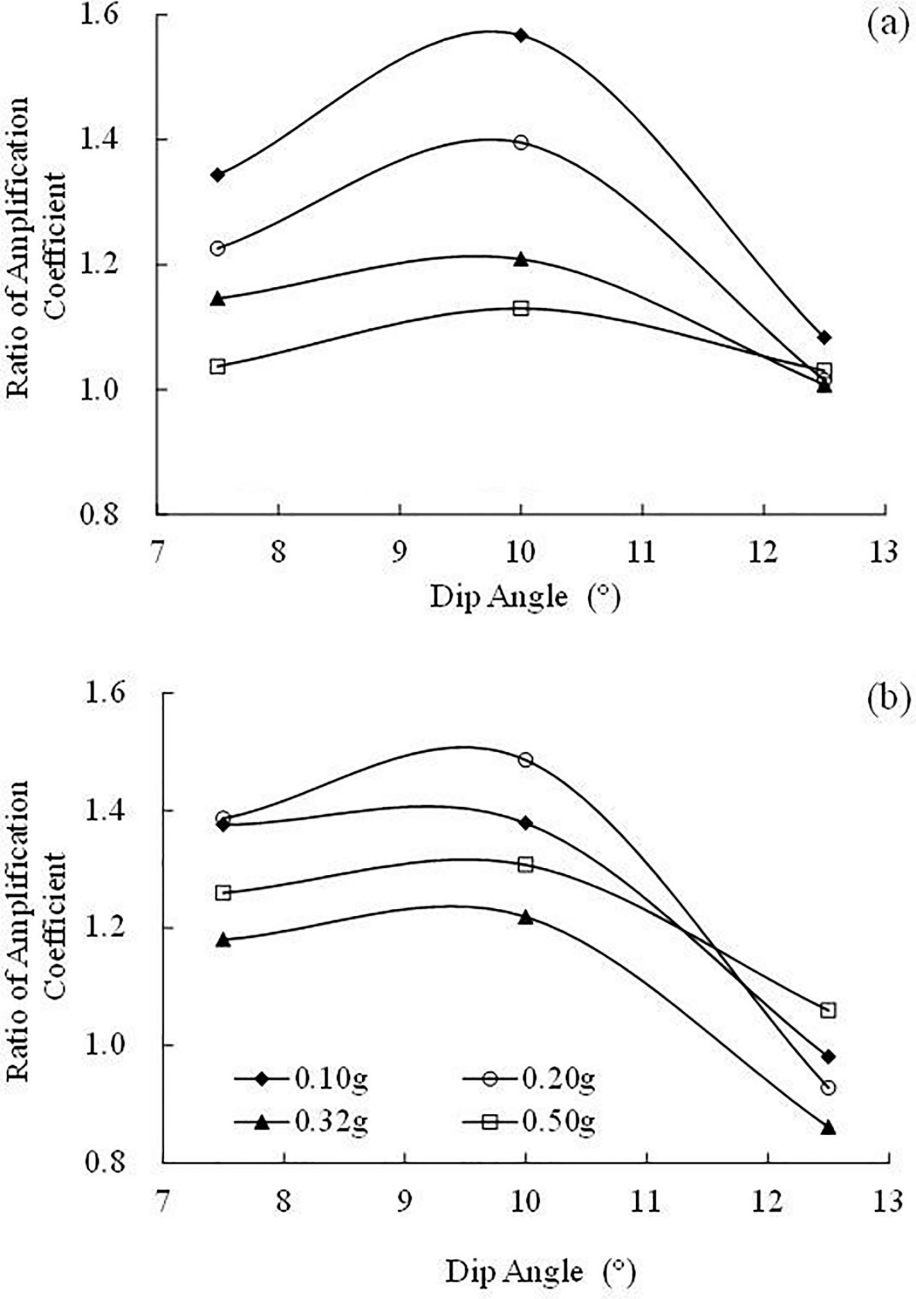
**Influence of dip angle on the ratio of amplification coefficient in the strike:** (a) under the El Centro earthquake record and (b) under the Wenchuan earthquake record. Ratio of acceleration amplification coefficient is defined as the ratio between the amplification coefficients of inclined layered site and the amplification coefficients of horizontal layered site. Less than 1 implying the acceleration amplification of the inclined layered site is weaker than horizontal layered site, otherwise, implying stronger than horizontal layered site.

In the strike, as the ratio of the Fourier spectrum is close to 1, the influence of dip angle on the 0–23 Hz frequency components can be ignored. In the frequency band of 23–40 Hz, the ratio of the Fourier spectrum of the 10° inclined layered site increases with increasing frequency, however, the ratios of the Fourier spectra of the 7.5° and 12.5° inclined layered sites decrease, and the reduction of the 7.5° inclined layered sites is slightly larger than that of the 12.5° inclined layered site. The above analysis shows that the dip angle has almost no influence on frequency components within 0–23 Hz. In the frequency band of 23–40 Hz, the frequency components of the 10° inclined layered site were amplified, whereas the frequency components in the 7.5° and 12.5° inclined layered sites were weakened, the reduction in the 12.5° inclined layered site is more serious than that in the 7.5° inclined layered site. The influence of dip angle on the Fourier spectrum conforms to the influence of dip angle on acceleration response in the strike, as shown in Fig 16. Fig 16 shows that the amplification effect of the 10° inclined layered site is larger than that of the 7.5° and 12.5° inclined layered sites. It implies that the 10° inclined layered site occurs stronger dynamic response compared with 7.5° and 12.5° inclined layered sites.

It is observed from Figs 14(C) and 15(C) that the influence of dip angle on frequency components in the vertical direction is negligible. The ratio of the Fourier spectra between the inclined layered sites and the horizontal site fluctuated symmetrically approximately 1.

### Effect of dip angle on response spectrum

**Fig 17:**
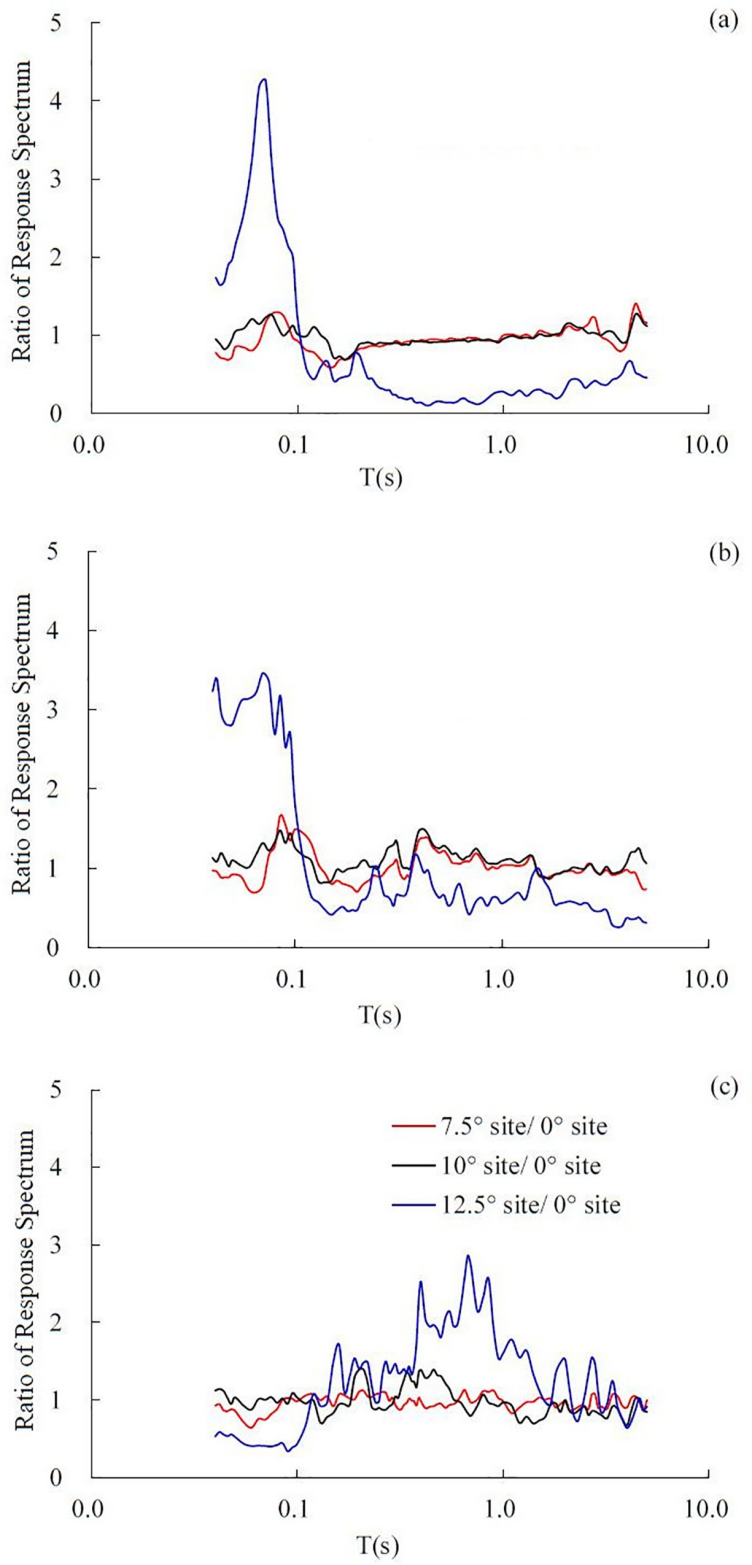
The ratio of the response spectrum under the 0.1 g El Centro earthquake wave in different directions, (a) in the dip direction, (b) in the strike and (c) in the vertical direction.

The ratio of the response spectrum was defined as the ratio of the response spectrum amplitude of gravel soil in the inclined layered site to that of gravel soil in the horizontal layered site. The ratio of the response spectrum represents the amplification effect on the response spectrum of the dip angle. All response spectra were calculated using the data collected from the top accelerators in the gravel soil layer, i.e., 5# accelerator for the horizontal layered site, 21# for the 7.5° inclined site, 4# for the 10° inclined site and 11# for the 12.5° inclined site. The ratio of the response spectrum of the X, Y, and Z directions under the 0.4 g El Centro earthquake record was calculated, as shown in Fig 17.

In the dip direction, strike and vertical directions, when the dip angle is 7.5° or 10°, the ratio of the response spectrum is near 1. When the dip angle is 12.5°, in the dip direction and strike, the amplification effect of the response spectrum is obvious in the short period (*T* ≤ 0.1 s). Conversely, when *T* > 0.1 s, the ratio of the response spectrum decreases to less than 1 rapidly, especially in the dip direction, the ratio of the response spectrum then keeps a low value, which indicates that when *T* > 0.1 s, the response spectrum amplitude of the 12.5° inclined layered site is relatively lower than that of the horizontal layered site in the dip direction and strike. In the vertical direction, it can be seen that the ratio of the response spectrum remains approximately 1 when the dip angle is 7.5° and 10°. It indicates that in the vertical direction, the influence of the dip angle on the response spectrum in the 7.5° and 10° sites is not obvious. However, with regard to the 12.5° inclined layered site, the response spectrum will be obviously amplified when *T* is approximately 0.8 s, and the ratio of the response spectrum reaches its peak value of 2.86 at *T* = 0.68 s.

### Amplification effect in the slope direction

**Fig 18:**
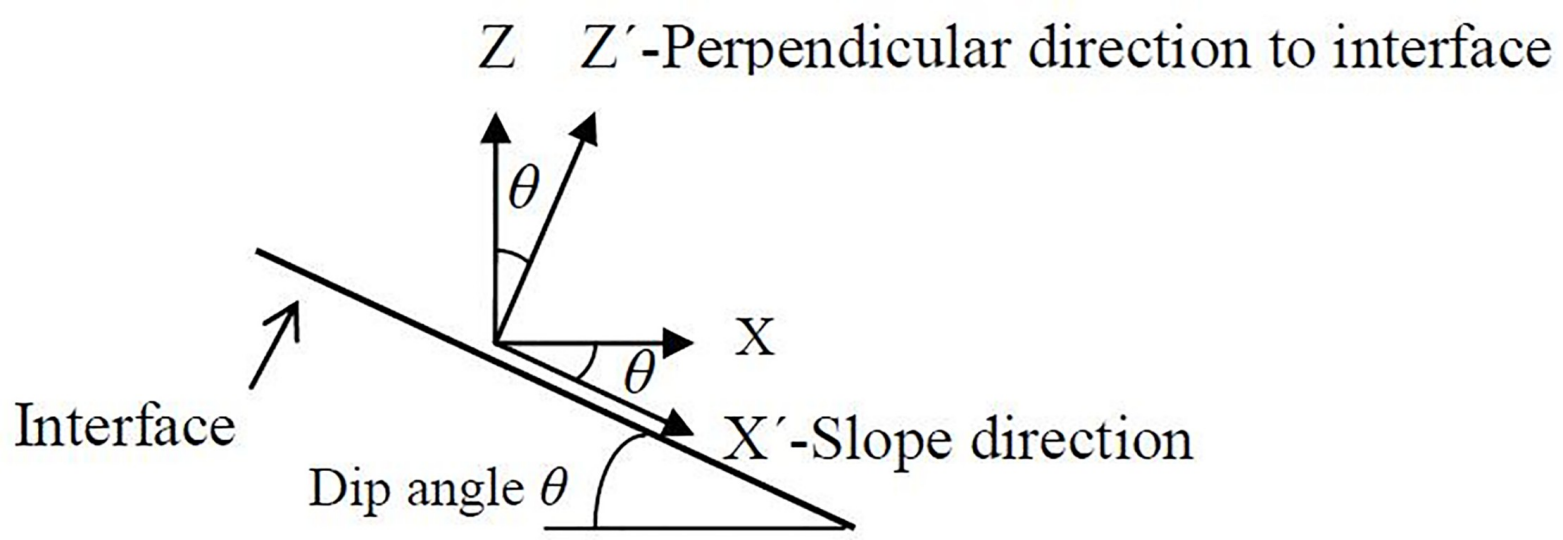
Illustration of coordinate transformation.

For the slopes that are prone to sliding along the interface, the dynamic response study in the slope direction and the direction perpendicular to the interface is significant for analysing the seismic failure mechanism. Here the “slope direction” is defined as the potential sliding direction along the interface, as shown in Fig 18. In the above analysis, the vertical direction is defined as the direction perpendicular to the horizontal surface. In the following analysis, the discussion will proceed based on the coordinate XˊYˊZˊ that rotates clockwise by a degree of *θ*, here *θ* is the dip angle of the interface, as shown in Fig 18. In the new coordinate system, the Zˊ-axis is perpendicular to the interface, the Xˊ-axis is in the slope direction, which is parallel to the interface, and the Yˊ-axis is the same as the Y-axis in the above analysis.

According to the transformation relationship shown in Fig 18, the transformation matrix between the XYZ coordinate system and the XˊYˊZˊ coordinate system is given by:

$$\left[\begin{array}{c} X^{\prime }\\ Y^{\prime }\\ Z^{\prime } \end{array}\right]=\left[\begin{array}{ccc} \cos\theta & 0 & -\sin\theta \\ 0 & 1 & 0\\ \sin\theta & 0 & \cos\theta \end{array}\right]\left[\begin{array}{c} X\\ Y\\ Z \end{array}\right]$$ (3)

**Fig 19:**
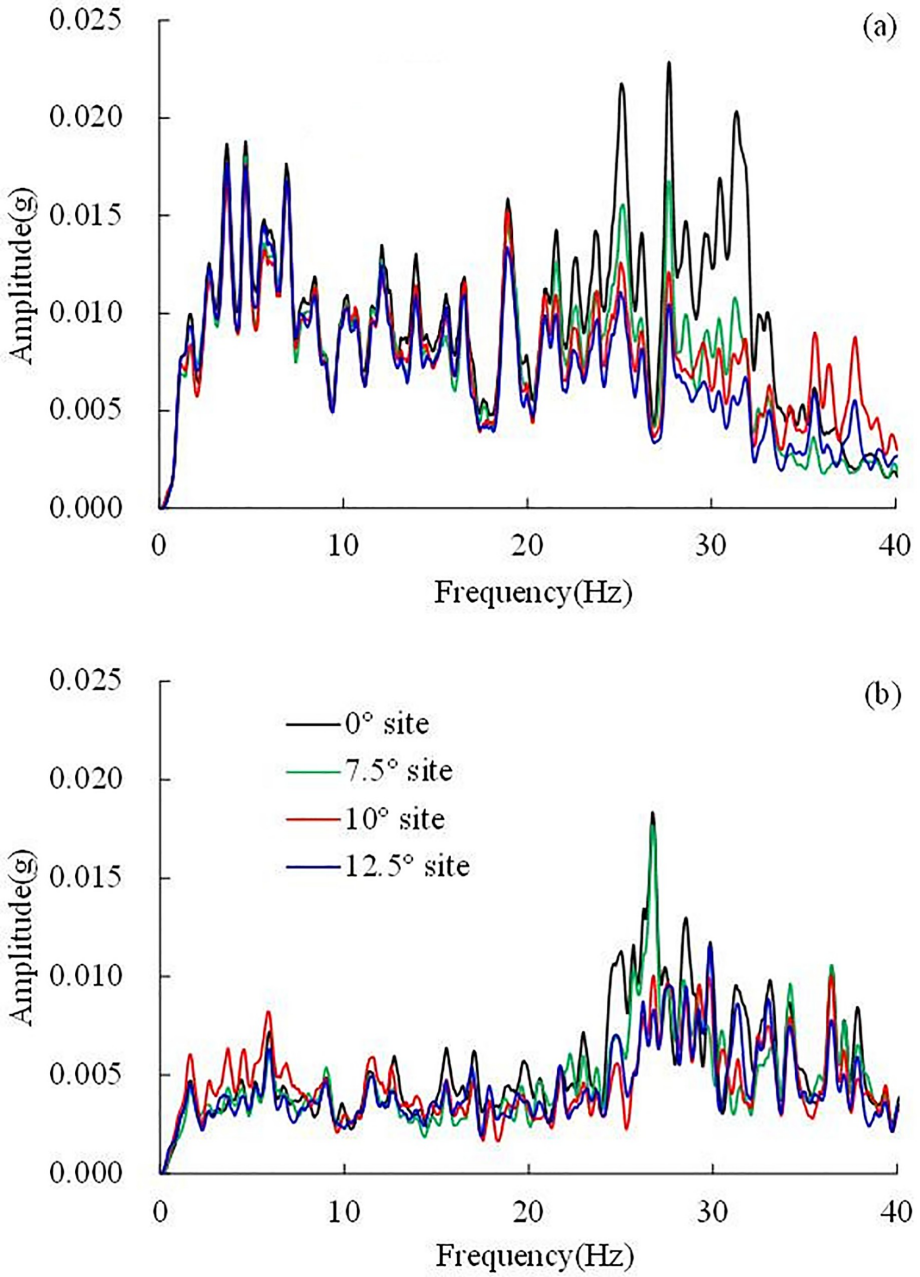
**The Fourier spectra under 0.1 g El Centro earthquake wave:** (a) in the slope direction, (b) in the direction perpendicular to the interface.

**Fig 20:**
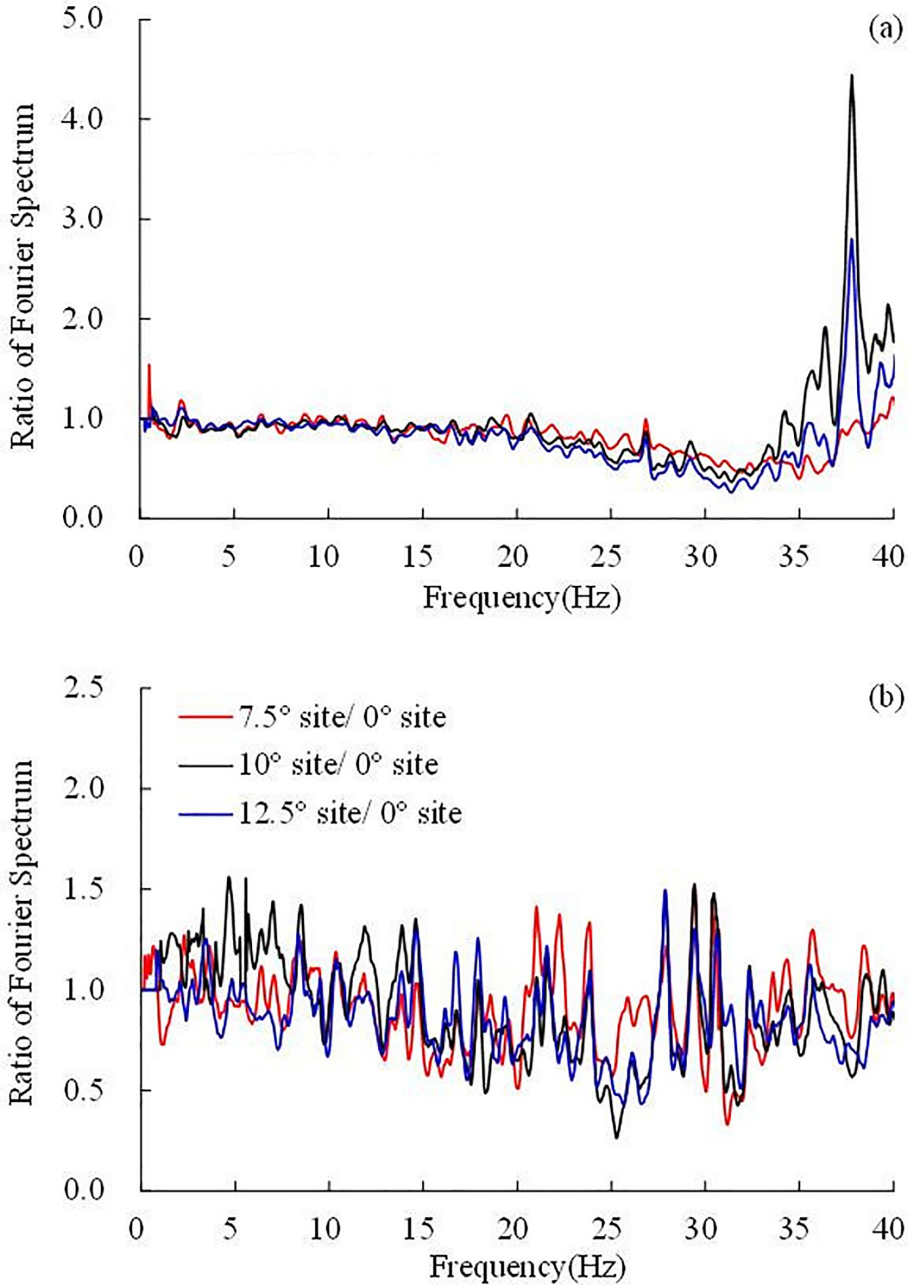
**Ratio of the Fourier spectra under 0.1 g El Centro earthquake wave:** (a) in the slope direction, (b) in the direction perpendicular to the interface.

The acceleration records of the XYZ axis can be projected onto the XˊYˊZˊ axis according to the transformation matrix. The Fourier spectra in the gravel soil layer of the four layered sites with dip angles of 0°, 7.5°, 10° and 12.5° under the 0.10 g El Centro earthquake wave were calculated. The Fourier spectra in the slope direction are shown in Fig 19(A), and the Fourier spectra in the direction perpendicular to the interface are shown in Fig 19(B). To indicate the influence of the dip angle on the frequency components, the horizontal site was selected as a reference, and the ratio of the Fourier spectrum is shown in Fig 20(A) and 20(B).

In the slope direction, the dip angle of the inclined layered site has a slight influence on the 0–20 Hz frequency components. The 20–35 Hz frequency components were weakened, whereas the 35–40 Hz frequency components were amplified, especially the frequency component of 38 Hz. In the 10° inclined layered site, 12.5° inclined layered site, and 7.5° inclined layered site, the ratio of the 38 Hz frequency component reached 4.5, 2.8, and 1, respectively, which cannot be explained according to the current state of knowledge.

As seen in Fig 19(B) and Fig 20(B), in the direction perpendicular to the interface, the dip angle has a slight influence on the frequency component, the ratio of the Fourier spectrum fluctuates in the range of 0.5–1.5, the frequency components near 15–40 Hz will be slightly weakened at the inclined layered site compared to the horizontal site. This phenomenon may be explained by the fact that the response in the perpendicular direction is the co-action of the earthquake wave in both the dip direction and vertical direction. The 18–40 Hz frequency components of the inclined layered sites were weakened by the inclined interface, and the vertical frequency components in the vertical direction are almost not dissipated by the site, which results in the reduction of frequency components near 15–40 Hz.

**Fig 21:**
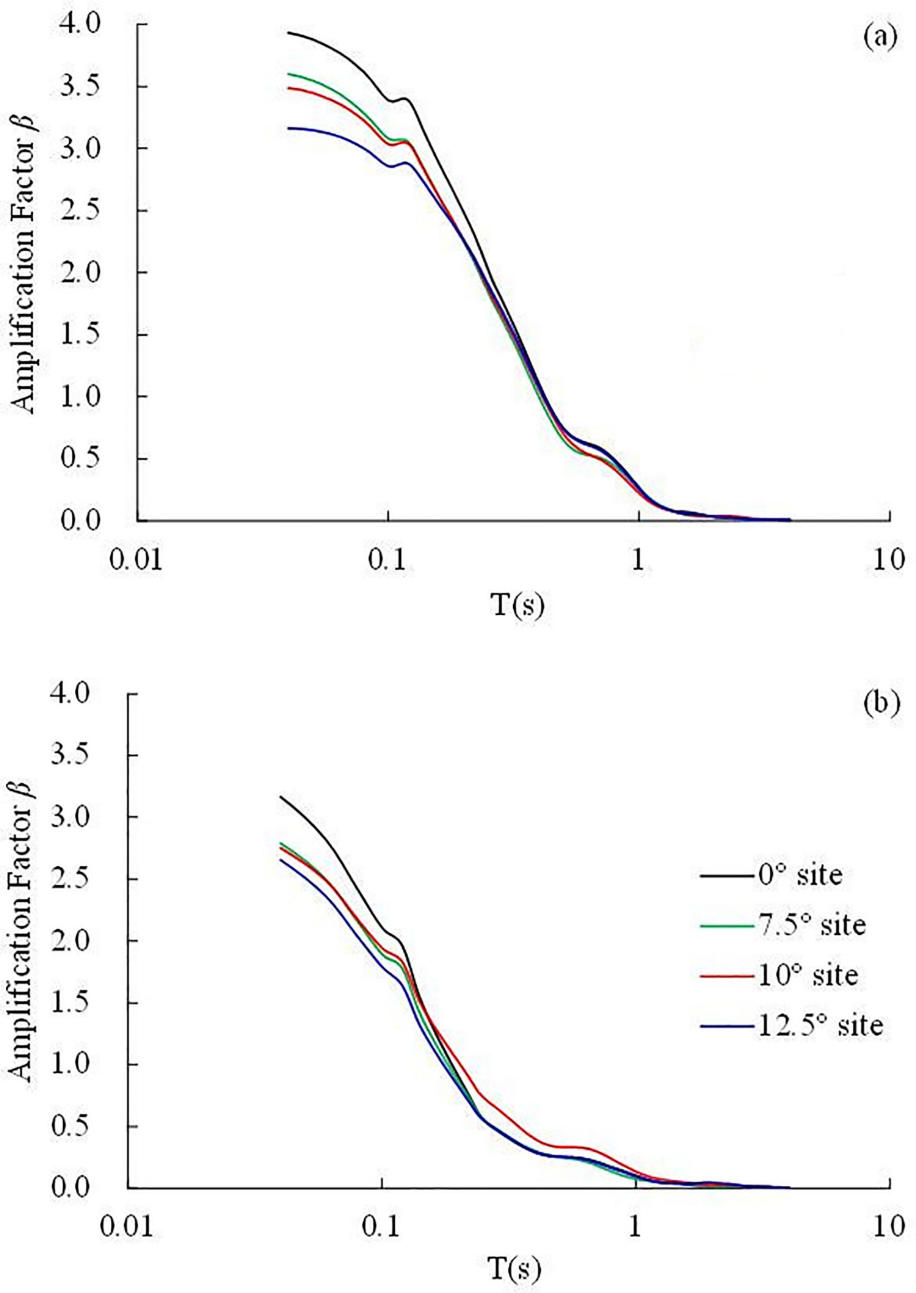
**The response spectra under the 0.1 g Kobe earthquake wave:** (a) in the slope direction, (b) in the direction perpendicular to the interface.

The acceleration response spectrum with a 5% damping ratio under the 0.10 g Kobe earthquake wave were calculated. The response spectra in the slope direction and the direction perpendicular to the interface are illustrated in Fig 21(A) and Fig 21(B). All data used to compute the response spectrum were measured by the top accelerometers (i.e., 5#, 21#, 4# and 11#).

It is easy to see from Fig 21(A) and Fig 21(B) that in the slope direction, the response spectra decrease with increasing dip angle in the period range of 0.02–0.105 s. In the period range of 0.105–0.13 s, the response spectra amplitude of the inclined layered site with dip angles 7.5°, 10° and 12.5° are very close, and all are less than that of the horizontal layered site. In the period range of 0.13–4.00 s, the response spectrum amplitudes of the inclined layered site and horizontal site are almost the same, that is to say, the influence of dip angle (≤12.5°) on the structures with periods of 0.13–4.00 s can be ignored.

In the direction perpendicular to the interface, the response spectra decrease with increasing dip angle in the period range of 0.02–0.11 s. In the period range of 0.11–1.00 s, the response spectrum amplitudes of the inclined layered site are close, and all are less than that of the 10° inclined layered site. In the period range of 1.00–4.00 s, the response spectrum curves almost coincide, namely the dip angle (≤12.5°) has little influence on the dynamic response of constructions with a period of 1.00–4.00 s.

It is worth noting that slope failure is the co-action result of acceleration both in the slope direction and the direction perpendicular to the interface. In practice, cases exist in which the slopes slide along the slope face with a small dip angle. For example, the landslide involving sliding failure along the slope face with a dip angle of 3–10° in the northeast PanZhihua airport in southwest China [31]. This section aims primarily at the study of small dip angle inclined layered sites. The findings in this paper may contribute to the investigation of slope failure mechanism under seismic excitation.

## Discussion

The differences and similarities of spectral characteristics between inclined layered sites and horizontal layered sites were compared in this paper using large scale shaking table tests, and the corresponding results were quantitatively analysed. The largest dip angle of the inclined layered sites in this model test was just 12.5°, so the model site should be defined as a small dip angle inclined layered site. Because some obtained results in this paper cannot be explained according to the current state of knowledge, whether the test result is suitable for larger dip angle inclined layered sites (i.e., *θ* > 12.5°) requires further study, and the research results in this paper require further practical verification and complement.

## Conclusions

According to the analyses of the test results, the following conclusions can be drawn.

1. In the horizontal layered site, the 13–40 Hz frequency components are amplified on a large scale in the horizontal direction, while hardly change in the vertical direction. The ratio of the Fourier spectrum in the gravel soil layer is larger than the soft rock layer. The amplification factor *β* of the gravel soil layer is larger than the soft rock layer and hard rock layer.
2. In the inclined layered site, the 18–40 Hz frequency components were weakened in the dip direction, the 23–40 Hz frequency components of the 10° site are amplified, while weakened in the 7.5° and 12.5° sites in the strike. In the dip direction and strike, the response spectra are not influenced by dip angle in the 7.5° and 10° sites, while obviously amplified when *T* ≤ 0.1 s and weakened rapidly when *T* > 0.1 s in the 12.5° site.
3. In the slope direction, the 20–35 Hz frequency components are weakened, while the 35–40 Hz frequency components are amplified. The response spectra amplitude of the 7.5°, 10° and 12.5° sites are close, and all are less than the horizontal layered site in the period range of 0.105–0.130 s and equal to the horizontal layered site in the period range of 0.13–4.00 s.
4. In the direction perpendicular to the interface, the response spectrum decreases with increasing dip angle in the period range of 0.02–0.11 s. In the period range of 0.11–1.00 s, the response spectrum amplitudes of the horizontal layered site, 7.5° and 12.5° sites are close, and all are less than the 10° site.

## Supporting information

S1 Data Input seismic wave. (ZIP)

S2 Data Recorded acceleration time history. (ZIP)

S3 Data Analysis data. (ZIP)
